# Inhibition of Xanthine Oxidoreductase Enhances the Potential of Tyrosine Kinase Inhibitors against Chronic Myeloid Leukemia

**DOI:** 10.3390/antiox9010074

**Published:** 2020-01-15

**Authors:** Marta Romo-González, Sara Moreno-Paz, Violeta García-Hernández, Fermín Sánchez-Guijo, Ángel Hernández-Hernández

**Affiliations:** 1 Department of Biochemistry and Molecular Biology, Universidad de Salamanca, Plaza Doctores de la Reina, 37007 Salamanca, Spain; martarogo@usal.es (M.R.-G.); saramorenopaz@gmail.com (S.M.-P.); violeta_gh@usal.es (V.G.-H.); 2 IBSAL (Institute for Biomedical Research of Salamanca), 37007 Salamanca, Spain; ferminsg@usal.es; 3 Hematology Department, University Hospital of Salamanca, Paseo de San Vicente, 139, 37007 Salamanca, Spain

**Keywords:** chronic myeloid leukemia (CML), reactive oxygen species (ROS), BCR-ABL, xanthine oxidoreductase (XOR), allopurinol, tyrosine kinase inhibitors (TKIs), imatinib, nilotinib

## Abstract

Chronic myeloid leukemia (CML) is characterized by the expression of the oncogenic kinase BCR-ABL. Although tyrosine kinase inhibitors (TKIs) against BCR-ABL represent the standard therapeutic option for CML, resistances to TKIs can be a serious problem. Thus, the search for novel therapeutic approaches is still needed. CML cells show an increased ROS production, which is required for maintaining the BCR-ABL signaling cascade active. In line with that, reducing ROS levels could be an interesting therapeutic strategy for the clinical management of resistant CML. To analyze the therapeutic potential of xanthine oxidoreductase (XOR) in CML, we tested the effect of XOR inhibitor allopurinol. Here, we show for the first time the therapeutic potential of allopurinol against BCR-ABL-positive CML cells. Allopurinol reduces the proliferation and clonogenic ability of the CML model cell lines K562 and KCL22. More importantly, the combination of allopurinol with imatinib or nilotinib reduced cell proliferation in a synergistic manner. Moreover, the co-treatment arms hampered cell clonogenic capacity and induced cell death more strongly than each single-agent arm. The reduction of intracellular ROS levels and the attenuation of the BCR-ABL signaling cascade may explain these effects. Finally, the self-renewal potential of primary bone marrow cells from CML patients was also severely reduced especially by the combination of allopurinol with TKIs. In summary, here we show that XOR inhibition is an interesting therapeutic option for CML, which can enhance the effectiveness of the TKIs currently used in clinics.

## 1. Introduction

Chronic myeloid leukemia (CML) is a hematological malignancy originated from the chromosomal translocation t(9,22)(q34;q11) that produces the Philadelphia chromosome [1]. As a result of this translocation, the oncogenic kinase BCR-ABL is expressed. This constitutively active kinase is capable of turning on several signaling pathways, including PI3K/AKT, STAT5, MAPKs, allowing growth factor-independent cell proliferation and escape of apoptosis [2]. With the discovery of specific tyrosine kinase inhibitors (TKIs) against BCR-ABL, the therapy of CML changed dramatically from a dismal to a very favorable outcome [3]. However, primary or secondary resistance to these treatments is still a serious threat for CML patients [4], which justifies the search for novel therapeutic options.

Tumor cells show a higher level of reactive oxygen species (ROS) than healthy cells [5]. Leukemic cells are not an exception, and they also show an elevated level of ROS [6]. The experimental evidence suggests that increased ROS production can contribute to the progression of hematological malignancies [7,8]. Bearing this in mind, the modulation of intracellular ROS levels is now considered a potentially attractive option to treat cancer [9]. Tumor cells are more sensitive than healthy cells to pro-oxidant treatments, which would provide a therapeutic window for oxidative substances [10]. The alternative option, reducing ROS levels, might also be valid since the increased production of ROS contributes to the proliferation of cancer cells [6,11].

NADPH oxidases are the only cellular system specialized in the production of ROS; besides, these enzymes are quantitatively the most important source of ROS after the mitochondria [12], and they are deeply involved in the control of redox signaling [13]. There is increasing evidence suggesting the implication of NADPH oxidases in the upregulation of ROS production in leukemic cells [6].

It has long been known that the transformation activity of some oncogenes, such as NRAS depends on the excessive ROS production through NADPH oxidases [14]. BCR-ABL also contributes to the increase of ROS in CML cells [15], which seems to be required for cell transformation and growth [16]. The upregulation of metabolism [17], the overproduction of ROS by the mitochondria, [15] and by NADPH oxidases [18] seem to be the main driving causes of oxidative stress in CML cells. In a previous investigation, we analyzed the use of NADPH oxidases as therapeutic targets in CML [19]. Our results showed that NADPH oxidase ROS production is required for maintaining the BCR-ABL signaling cascade. We showed that diminishing ROS levels through the inhibition or silencing of NADPH oxidases reduces the proliferation and clonogenic ability of CML cells. Moreover, the combination of TKIs with NADPH oxidases inhibitors showed a strong synergistic effect, which makes NADPH oxidases a very promising therapeutic target against CML.

Xanthine oxidoreductase (XOR) participates in the catabolism of purines [20], catalyzing the oxidation of hypoxanthine to xanthine and xanthine to uric acid. In mammals, the enzyme is initially synthesized as xanthine dehydrogenase (XDH), and it can be converted to xanthine oxidase (XO) by proteolysis or by oxidation [20]. Despite the structural differences and substrate specificity, both forms of the enzyme can produce ROS as a secondary product. XDH would mainly lead to superoxide (O_2_^–^) production, while XO would eventually generate hydrogen peroxide (H_2_O_2_) [20]. Therefore, XOR represents another important cellular source of ROS. Interestingly, it has been demonstrated that NADPH oxidases can be activated by XOR [21]. This functional connection leads us to hypothesize that targeting XOR might be an interesting therapeutic approach against CML.

XOR chemical inhibition has been used in the prevention and treatment of gout for more than half a century [22], which guarantees the safety of long-term treatment with XOR inhibitors in humans. In addition, it comes at an extremely low health cost compared to most targeted drugs currently under development. Our results show that allopurinol reduces the proliferation, clonogenic ability, and ROS levels in the CML model cell lines. More importantly, allopurinol enhances the inhibitory effect on cell proliferation of two of the TKIs most currently employed in the first-line treatment (imatinib and nilotinib) in a synergistic manner. In addition, the clonogenic ability, and the intracellular ROS levels decreased more sharply upon the co-treatment arms. These results correlate with the reduction of intracellular ROS levels and with the downregulation of the BCR-ABL signaling cascade. The feasibility of this therapeutic strategy also holds true for primary bone marrow cells from CML patients. In summary, our results support the use of XOR inhibitors as a therapeutic strategy against CML, which could enhance the effect of the TKIs, and therefore the clinical treatment options.

## 2. Materials and Methods

### 2.1. Cell Culture

K562 and KCL22 cells were purchased from Sigma Aldrich (Madrid, Spain) and DSMZ (Braunschweig, Germany), respectively. Cell lines were tested for *Mycoplasma* spp. contamination prior to use with the PlasmoTest detection kit (InvivoGen, Toulouse, France, cat #rep-pt1). Cell lines were grown in 10% FBS-supplemented RPMI medium plus 100 U/mL penicillin, 100 U/mL streptomycin, and 2 mmol/L l-glutamine at 37 °C and 5% CO_2_. Cell culture reagents were from Biowest (VWR, Madrid, Spain). Bone marrow mononuclear cells (BM-MNC) from chronic phase CML patients at diagnosis were obtained at the University Hospital of Salamanca. In all cases, informed consent (as approved by the local Ethics Committee, protocol number 2014/02/38) was obtained from each patient.

### 2.2. Cell Proliferation Analysis

Cell proliferation was monitored by MTT assay (3-(4,5-dimethylthiazol-2-yl)-2,5-diphenyltetrazolium bromide), and by cell counting in the presence of trypan blue, as before [19,23]. Cells were washed with PBS, resuspended in 0.5 mg/mL MTT, and incubated at 37 °C, for 75 min in the dark. Afterward, cells were washed with PBS, resuspended in DMSO and the absorbance at 570 nm was measured. MTT and DMSO were from Sigma Aldrich (Madrid, Spain).

### 2.3. Analysis of Drug Interactions

Drug interaction was analyzed by the median-effect method as described by Chou-Talalay [24], as it has been extensively endorsed in the scientific literature [25,26,27,28,29]. The combination index (CI), calculated with the CalcuSyn software (Biosoft, Cambridge, UK), establishes the interaction between drugs: Synergy (CI < 1), additivity (CI = 1), or antagonism (CI > 1).

### 2.4. Cell Viability Analysis

Cell viability was analyzed by flow cytometry after staining with an Annexin V-PE/7-aminoactinomycin (7-AAD) detection kit (Immunostep, Salamanca, Spain) per the manufacturer’s instructions.

### 2.5. Colony Forming Unit Assays

Cell clonogenic capacity was analyzed by colony-forming unit (or CFU) assays in semisolid methylcellulose medium as previously described [30]. K562 and KCL22 cells or primary bone marrow mononuclear cells (BM-MNC) from CML patients were treated with two different TKIs (either imatinib or nilotinib), allopurinol, and their combinations in RPMI medium for 48 h. Cells were then washed with PBS and 500 K562 and KCL22 cells, or 12500 BM-MNC cells were resuspended in 500 μL of “HSC-CFU-basic” or “HSC-CFU-complete w/o Epo,” respectively (Miltenyi Biotec; Madrid, Spain) and seeded on a culture plate. Cells were grown at 37 °C and 5% CO_2_, and colonies were counted by blinded scoring at day 7 for K562 and KCL22 cells, and at day 14 for primary samples. CFU identification and counting were performed according to the criteria previously described [31].

### 2.6. Detection of Intracellular ROS Levels

Intracellular ROS levels were detected with 2′,7′-dichlorofluorescein diacetate (DCFDA) as described before [19,23]. Cells were stained with 10 μM DCFDA (Sigma Aldrich, Madrid, Spain) at 37 °C for 30 min in the dark and washed twice with PBS. ROS levels were detected by flow cytometry.

### 2.7. Immunoblotting

Cells were resuspended in MLB lysis buffer (25 mM HEPES, pH 7.5, 150 mM NaCl, 1% Igepal, 10% glycerol, 10 mM MgCl_2_, 1 mM EDTA, 25 mM NaF, 1 mM Na_2_VO_4_, plus proteinase inhibitors) and incubated on ice for 20 min. Soluble protein extract was obtained after centrifugation at 20,000× *g* 15 min. Proteins were then separated by dodecyl sulfate-polyacrylamide gel electrophoresis (SDS-PAGE) and transferred onto polyvinylidene fluoride (PVDF) membranes. Quantification of bands was performed by densitometry analysis as previously described [19,23], and by fluorescently labeled secondary antibodies with a ChemiDoc MP device (BIO-RAD, Madrid, Spain). Anti-phospho-c-ABL (pY412), anti-c-ABL, and anti-STAT5 were from Santa Cruz Biotechnology (Santa Cruz, CA, USA). Anti-phospho-STAT5 (pY694) was purchased from BD Bioscience (Madrid, Spain), and Anti-GAPDH was supplied by Sigma Aldrich (Madrid, Spain).

### 2.8. Statistical Analysis

Results are shown as the mean ± standard error. Student’s *t*-test and ANOVA test (Tukey for unequal variances and Games–Howell for equal variances as post hoc tests) were used for two-group and multiple group comparisons respectively. Excel and SPSS software were used for the analysis. Differences were considered statistically significant when *p* < 0.05 (*), *p* < 0.01, (**), and *p* < 0.001 (***).

## 3. Results

### 3.1. The XOR Inhibitor Allopurinol Inhibits K562 Cells Proliferation

**Figure 1:**
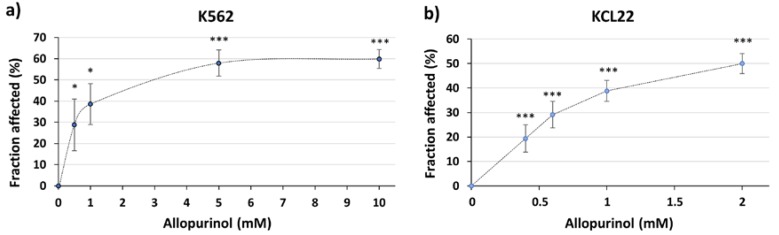
Allopurinol reduces the proliferation of the K562 and KCL22 cells. Cells were treated with different concentrations of allopurinol for 48 h. Proliferation was analyzed by MTT assay. (**a**) Results show the fraction affected or percentage of inhibition with respect to control in K562 cells (*n* = 3). (**b**) Fraction affected or percentage of inhibition with respect to control in KCL22 cells (*n* = 6). * *p* < 0.001 and *** *p* < 0.05 reflect significant differences with respect to control.

Allopurinol is a hypoxanthine isomer that can inhibit XOR, used for the treatment of gout and other hyperuricemia related conditions [32]. To test the feasibility of using XOR as a therapeutic target in CML, we used two model BCR-ABL-positive cell lines (K562 and KCL22). As shown in Figure 1, treatment with allopurinol significantly reduced cell proliferation (Figure 1), with an IC_50_ of 3.35 mM and 1.80 mM for K562 and KCL22 cells, respectively.

### 3.2. Allopurinol and TKIs Inhibits K562 and KCL22 Cells Proliferation in a Synergistic Manner

**Figure 2:**
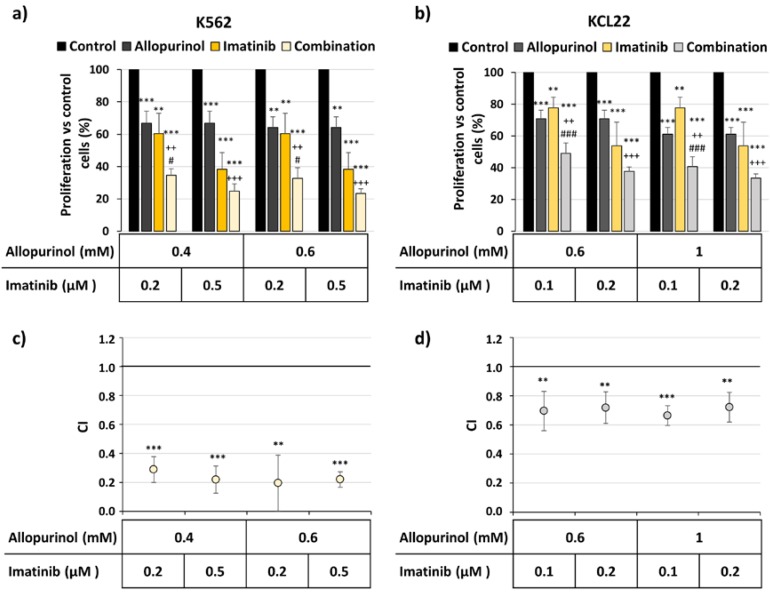
The combination of imatinib and allopurinol reduces the proliferation of the K562 and KCL22 cells in a synergistic manner. K562 and KCL22 cells were treated with different concentrations of imatinib, allopurinol or their combination for 48 h. Proliferation was analyzed by MTT assay and the combination indexes (CI) were calculated as described in the Methods section. (**a**) K562 cells proliferation with respect to control (*n* = 4). (**b**) KCL22 cells proliferation with respect to control (*n* = 5). Significant differences: *** *p* < 0.001, ** *p* < 0.01 with respect to control; ^+++^ *p* < 0.001, ^++^ *p* < 0.01 with respect to allopurinol-treated cells; ^###^ *p* < 0.001, ^#^ *p* < 0.05 with respect to imatinib-treated cells. (**c**) Mean CI values for the drug combinations tested in K562 cells (*n* = 4). (**d**) Mean CI values for the drug combinations tested in KCL22 cells (*n* = 5). *** *p* < 0.001 and ** *p* < 0.01 reflect significant differences with respect to CI value 1.

Bearing in mind that CML cells are sensitive to allopurinol treatment (Figure 1), we next combined imatinib and allopurinol (Figure 2). The inhibition of cell proliferation was significantly more pronounced with the combination in both K562 (Figure 2a) and KCL22 cells (Figure 2b). Moreover, the analysis of drug interaction showed CIs significantly below 1, thus reflecting a strong synergistic effect of the allopurinol plus imatinib combinations in both cell lines (Figure 2c,d).

**Figure 3:**
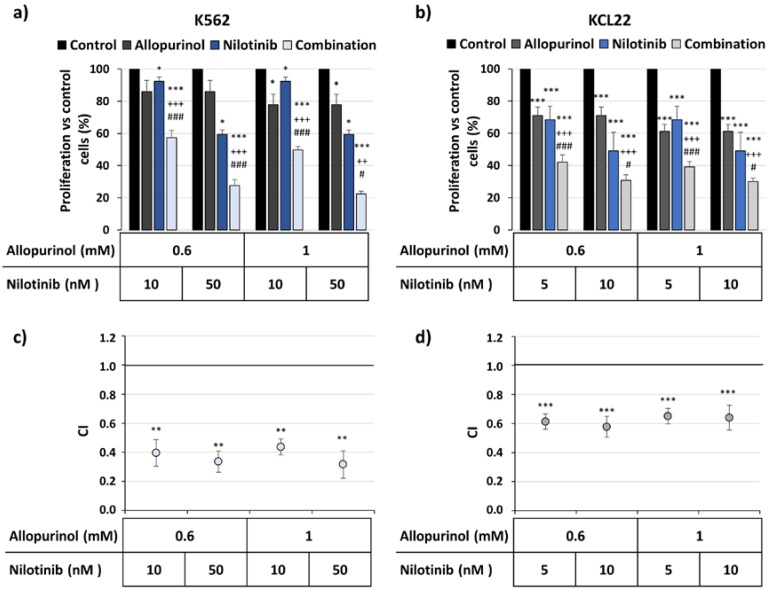
The combination of nilotinib and allopurinol reduces the proliferation of the K562 and KCL22 cells in a synergistic manner. K562 and KCL22 cells were treated with different concentrations of nilotinib, allopurinol, or their combination for 48 h. Proliferation was analyzed by MTT assay and the combination indexes (CI) were calculated as described in the Methods section. (**a**) K562 cells proliferation with respect to control (*n* = 3). (**b**) KCL22 cells proliferation with respect to control (*n* = 5). Significant differences: *** *p* < 0.001 with respect to control, * *p* < 0.05; ^+++^ *p* < 0.001, ^++^ *p* < 0.01 with respect to allopurinol-treated cells; ^###^ *p* < 0.001, ^#^ *p* < 0.05 with respect to nilotinib-treated cells. (**c**) Mean CI values for the drug combinations tested in K562 cells (*n* = 3). (**d**) Mean CI values for the drug combinations tested in KCL22 cells (*n* = 5). *** *p* < 0.001 and ** *p* < 0.01 reflect significant differences with respect to CI value 1.

To test this further, the combination of a second-generation BCR-ABL inhibitor, nilotinib, with allopurinol was also tested. In agreement with the results described above, the combination reduced the proliferation more strongly than the individual treatments (Figure 3a,b). Moreover, the analysis of the drug interaction supports the synergism between both drugs, as the CI was below 1 (Figure 3c,d).

**Figure 4:**
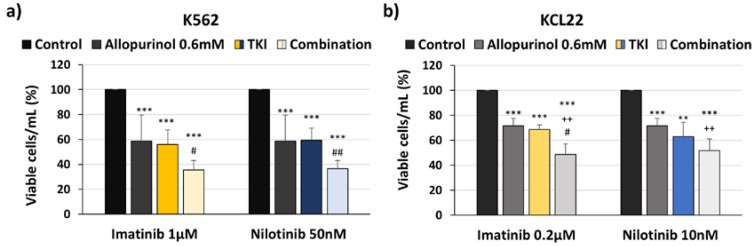
Allopurinol and tyrosine kinase inhibitor (TKI) combination reduces cell number more strongly than single agents. K562 and KCL22 cells were treated with TKIs (imatinib or nilotinib), allopurinol, or their combinations for 48 h. The number of viable cells was counted by trypan blue exclusion. (**a**) Number of viable K562 cells with respect to control (*n* = 5). (**b**) Number of viable KCL22 cells with respect to control (*n* = 5). Significant differences: *** *p* < 0.001, ** *p* < 0.01 with respect to control; ^++^ *p* < 0.01 with respect to allopurinol-treated cells; ^##^ *p* < 0.01, ^#^ *p* < 0.05 with respect to TKI-treated cells.

In line with the results described in Figure 2 and Figure 3, a stronger reduction of viable cell numbers by the co-treatment arms with respect to single arms was also observed by cell counting with trypan blue exclusion (Figure 4).

### 3.3. Allopurinol and TKIs Co-Treatment Induces Cell Death More Efficiently Than Individual Treatments

**Figure 5:**
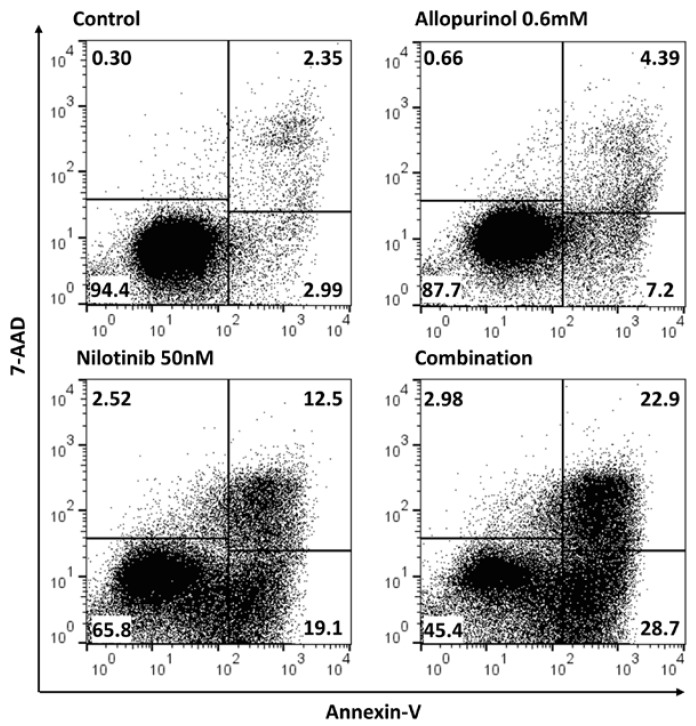
The combination of nilotinib and allopurinol induces K562 cell death more efficiently than individual treatments. K562 cells were treated with 50 nM nilotinib, 0.6 mM allopurinol or their combination for 48 h. Cell viability was analyzed by flow cytometry staining with Annexin-V/7AAD: viable (Annex^−^/7AAD^−^), early (Annex^+^/7AAD^−^) and late (Annex^+^/7AAD^+^) apoptotic, and necrotic cells (Annex^−^/7AAD^+^). A representative flow cytometry diagram is shown (*n* = 5).

We next analyzed the effect on cell viability. While the single-treatment with each drug induced a subtle decrease in the percentages of viable cells, the most pronounced effect was observed upon the co-treatment arms (allopurinol + TKI) in both model cell lines (Figure 5 and Table 1 and Table 2). The combination induced a stronger decrease in the number of viable cells and in an increase of apoptotic cells (Figure 5, Table 1 and Table 2). Therefore, the addition of allopurinol to either imatinib or nilotinib induces cell death more efficiently than individual treatments.

### 3.4. Allopurinol and TKIs Combination Reduces K562 and KCL22 Cells Clonogenic Capacity

**Figure 6:**
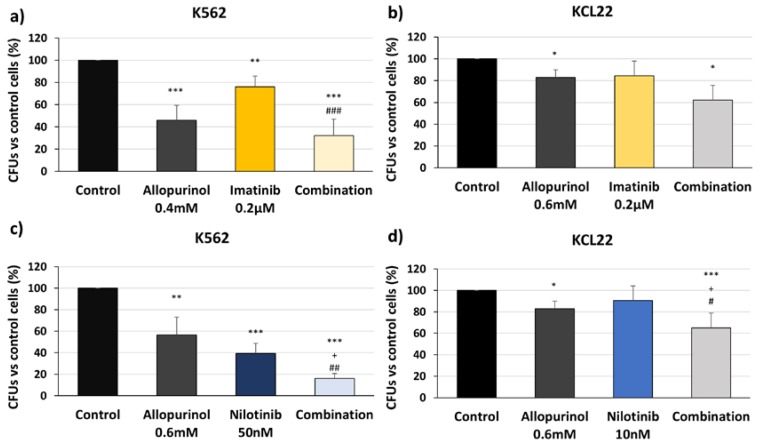
Allopurinol and TKI combination reduces the clonogenic capacity of the K562 and KCL22 cells. K562 and KCL22 cells were treated with TKIs (imatinib or nilotinib), allopurinol, or their combinations for 48 h. The clonogenic capacity after the treatments was analyzed in a semisolid medium by colony-forming unit (CFU) assays. (**a**) CFU number with respect to control in K562 treated with imatinib, allopurinol or their combination (*n* = 5). (**b**) CFU number with respect to control in KCL22 treated with imatinib, allopurinol or their combination (*n* = 4). (**c**) CFU number with respect to control in K562 treated with nilotinib, allopurinol or their combination (*n* = 5). (**d**) CFU number with respect to control in KCL22 treated with nilotinib, allopurinol or their combination (*n* = 4). Significant differences: *** *p* < 0.001, ** *p* < 0.01, * *p* < 0.05 with respect to control; ^+^ *p* < 0.05 with respect to allopurinol-treated cells; ^##^ *p* < 0.01, ^#^ *p* < 0.05 with respect to TKIs-treated cells.

The effect of anti-leukemic drugs on cell renewal capacity is an important aspect to analyze. This can be done through colony-forming unit assays. Allopurinol treatment reduced the clonogenic capacity of both K562 and KCL22 cells (Figure 6), thereby supporting again the potential use of allopurinol against CML. At the concentration used, both TKIs (imatinib and nilotinib) reduced the clonogenic ability of K562 cells (Figure 6a,c), despite the fact that no significant effect was seen in KCL22 cells. However, the combinations of allopurinol + TKI showed the most potent effect. Allopurinol + nilotinib co-treatment was the most effective treatment, leading to a significant decrease in colonies when comparing either to the control or to the single treatment (Figure 6c,d). This evidence, in line with the results described above, supports the benefit of adding allopurinol to TKIs.

### 3.5. Imatinib, Allopurinol, and Their Combination Reduce Intracellular ROS Levels

**Figure 7:**
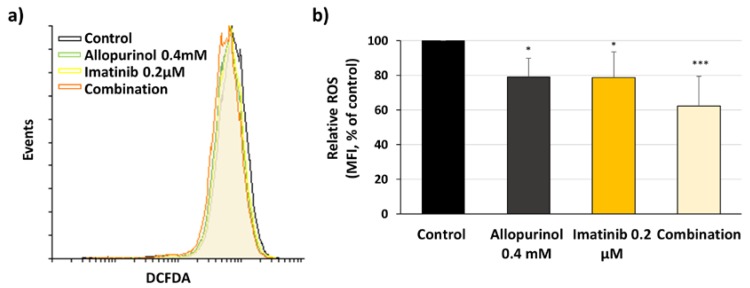
Imatinib, allopurinol, and their combination reduce intracellular reactive oxygen species (ROS) levels. K562 cells were treated with 0.2 μM imatinib, 0.4 mM allopurinol or their combination for 6 h. Intracellular ROS levels were analyzed by flow cytometry staining with 2′,7′-dichlorofluorescein diacetate (DCFDA). (**a**) A representative flow cytometry histogram is shown. (**b**) DCFDA mean fluorescence intensity with respect to control reflecting intracellular ROS levels is shown (*n* = 8). *** *p* < 0.001, * *p* < 0.05 reflects significant differences with respect to control.

ROS are important for CML progression, as they facilitate BCR-ABL signaling [19] and increase genetic instability which can eventually lead to progression into the blastic phase of the disease [17]. We reasoned that analyzing the intracellular ROS levels upon the different treatments could help to explain the effects described in the previous sections. In agreement with previous reports [19,33], imatinib treatment reduced the level of intracellular ROS (Figure 7). XOR inhibition with allopurinol also induced a significant reduction in the level of intracellular ROS. The combination of imatinib/allopurinol caused the strongest reduction with respect to the control (Figure 7). While individual treatments induce a decrease of 20%, the combination treatment almost reaches a 40% reduction, suggesting an additive effect of both agents regarding the reduction in ROS levels.

### 3.6. Imatinib, Allopurinol and Their Combination Attenuate BCR-ABL Signaling

**Figure 8:**
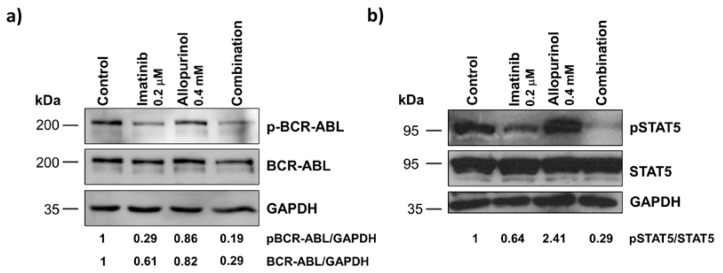
The combination of imatinib and allopurinol reduces the levels of phospho-BCR-ABL, BCR-ABL, and phospho-STAT5. K562 cells were treated with 0.2 μM imatinib, 0.4 mM allopurinol, or their combination for 6 h. The level of the phosphorylated-activated forms of BCR-ABL and STAT5 was analyzed by immunoblotting. The same membranes were stripped and reprobed to detect the total levels of these proteins. GAPDH was used as a loading control. Representative experiments are shown. (**a**) pBCR-ABL/BCR-ABL levels (*n* = 6). (**b**) pSTAT5/STAT5 (*n* = 4) levels.

The BCR-ABL signaling cascade upon the different treatments was analyzed next. By western blotting, we analyzed the level of activation of BCR-ABL itself, and also the level of activation of STAT5, a direct target of BCR-ABL, and a prominent driver of CML [34]. Imatinib treatment induced a decrease in both the level of activated BCR-ABL (phosphorylated form) and in the total protein levels (Figure 8a), which was in line with our previous results [19]. Interestingly, a very similar result was found upon allopurinol treatment (Figure 8a). The combination of both agents showed a significant decrease in the level of both, the activated form of BCR-ABL and in the total levels of the protein. Such a decrease in BCR-ABL could explain the results we have found: the inhibition of proliferation, an increase of cell death and a reduced clonogenic capacity.

When we analyzed STAT5, a characteristic hyper-activated target in CML cells, no differences were found regarding the global levels of this signaling protein. The phosphorylated active form of STAT5 was significantly reduced by imatinib treatment while allopurinol showed the opposite effect. However, the strongest reduction of STAT5 activation was observed upon the co-treatment arm (Figure 8b).

### 3.7. The Combination of Allopurinol and TKIs Reduces the Clonogenic Ability of CML Primary Cells

**Figure 9:**
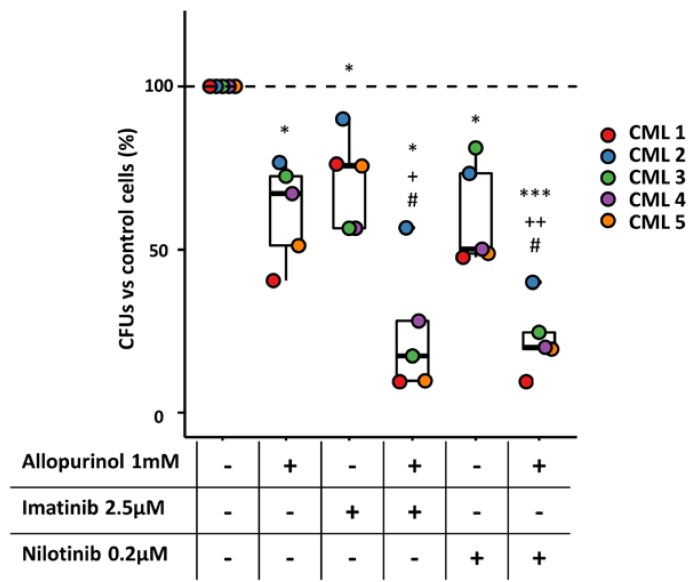
The combination of TKIs and allopurinol reduces the clonogenic capacity of primary chronic myeloid leukemia (CML) cells. BM-MNCs collected from CML patients were treated with TKIs (imatinib and nilotinib), allopurinol, or their combinations for 48 h. The clonogenic capacity after the treatments was analyzed in a semisolid medium by CFU assays. Relative colony numbers of BM-MNCs treated with TKI (imatinib or nilotinib), allopurinol, or their combination are shown. *** *p* < 0.001, * *p* < 0.05 with respect to control; ++ *p* < 0.01, + *p* < 0.05 with respect allopurinol-treated cells; # *p* < 0.05 with respect to TKI-treated cells. (*n* = 5).

Finally, to analyze the clinical potential of the combinations tested on cell lines, the clonogenic ability of BM-MNC from CML patients in the presence of the different treatments was studied. Interestingly, the results obtained were very similar to those described above for K562 and KCL22 cells (Figure 6). Allopurinol or TKIs individually reduced CFU numbers in all patients, but the strongest effect was observed upon the combinations, which led to a significant decrease in colonies when comparing either to the control or to the single drugs (Figure 9). These observations validate the results described above for CML cell lines, and more importantly, support the feasibility of adding allopurinol to increase the effect of TKIs on CML patients.

## 4. Discussion

Eukaryotic cells must cope with the continuous formation of ROS, derived from their aerobic metabolism. ROS have traditionally been considered harmful for cell physiology [35], however, over the last two decades, accumulated evidence supports the importance of a moderate production of ROS for the control of cellular signaling and gene expression [13]. It is accepted that an uncontrolled production of ROS is related to aging and to the development of degenerative diseases and cancer [6,11]. Cancer cells show an elevated level of ROS compared to healthy cells [36,37], a factor that may contribute to tumorigenesis and cancer progression by different mechanisms [38,39]. ROS can damage DNA, increasing the mutation rate [40], thus affecting epigenetic modifications [41], or modifying the activity of different transcription factors involved in cancer [42]. Finally, the transforming activity of several oncogenes, such as KRAS [14,43] or BCR-ABL [16] depends on the upregulated production of ROS.

Increasing ROS levels have been postulated as an attractive therapeutic strategy against cancer [10]. Reducing ROS levels could also be an appealing therapeutic approach, given the importance of ROS in sustaining tumor growth. However, the use of antioxidants in oncology is still a matter of debate that requires further evaluation [44]. Using antioxidants as a co-adjuvant therapy may reduce the toxic side effects produced by pro-oxidants drugs [45], while at the same time antioxidants can reduce the cytotoxicity of many chemotherapeutics.

An alternative to reducing ROS by the use of antioxidants is the inhibition of ROS production sources. This strategy might be more effective and specific if we knew the origin of ROS in tumor cells. NADPH oxidases are one of the main sources of ROS in the cell [12], and they may be an important source of ROS in cancer [46]. We have shown the importance of NADPH oxidase ROS production to maintain BCR-ABL signaling. Moreover, our results in vitro and in vivo show that NADPH oxidases are a potential therapeutic alternative against CML alone or in combination with TKIs [19].

The downregulation and upregulation of XOR activity have been related to different types of solid tumors [47], suggesting the importance of XOR produced ROS in tumorigenesis [48]. Besides, a higher XOR activity has been related with relapsed acute myeloid leukemia [49]. XOR has been used before as a tool to increase oxidative stress in tumor cells as a therapeutic strategy, both in hematological malignancies and solid tumors [48]. Here we have tested the alternative approach, the inhibition of XOR activity against CML. Our results show that allopurinol reduces the proliferation and the clonogenic ability of CML cells, suggesting the feasibility of targeting XOR in CML treatment. More importantly, allopurinol and TKI combination showed a prominent synergistic effect inhibiting CML cell line proliferation. The results obtained in bone marrow cells from CML patients also support the potential of allopurinol to increase the effect of TKIs. While allopurinol and TKIs individually reduced CFU numbers, the combinations showed the strongest effect. Therefore, XOR inhibition could be harnessed to increase the efficacy of the TKIs currently used in the clinic. Although the use of TKIs against CML is one of the best examples of molecular targeted therapy, primary or secondary TKI resistances are still a serious concern [4]. Searching alternatives to enhance TKI effect is a worthwhile endeavor. This would allow the use of lower TKIs doses, which would hamper the appearance of TKIs resistances. In this line, our results offer a very promising strategy by using the XOR inhibitor allopurinol in combination with BCR-ABL inhibitors. Another important aspect of our results is the fact that allopurinol has long been used for the prevention and treatment of gout, which guarantees the safety of human treatment, in addition to its reduced financial cost. Furthermore, many patients at diagnosis combine allopurinol with a TKI to prevent tumor lysis syndrome (TLS), although the former is usually subsequently discontinued [50].

The inhibition of BCR-ABL phosphorylation is the base of the TKIs’ mechanism of action. Here we show that allopurinol enhances the potential of TKIs to avoid BCR-ABL activation. This is probably one of the main molecular mechanisms explaining the effectiveness observed upon the combination. In line with our previous results [19], we suggest that a reduction in the intracellular ROS levels by XOR inhibition could hamper BCR-ABL signaling cascade activation. There is an interesting report showing that XOR can enhance NADPH oxidase activity by upregulating cytosolic calcium concentration, which would lead to a further increase in ROS [21]. Therefore, it is tempting to speculate that the effects of allopurinol against CML cells reported here, may well be due to the reduction of ROS production, not only by XOR but also by NADPH oxidases.

The inhibition of STAT5 has been suggested as a feasible therapeutic strategy against CML [51]. Upon co-treatment, we observed a significant decrease in STAT5 activation, which could contribute to the synergistic effect described here. However, allopurinol alone induced a notable increase in the levels of the active form of STAT5. The upregulation of STAT3 in myocardial cells upon allopurinol treatment, probably through JAK2 activation, has been previously described, [52]. In line with this report, we hypothesized that the upregulation of STAT5 activation by allopurinol could also be due to JAK2 activation. The inhibitory effect of the combined therapy on STAT5 activation could thus be explained by the ability of imatinib to inhibit STAT5 activation through JAK2 dependent and independent mechanisms [34]. A similar effect has been reported for the combination of PIM kinases inhibitors with TKIs [53], while PIM kinases inhibitors induced the upregulation of STAT5, the combination of these agents with TKIs led to a strong decrease in STAT5 activation. In addition, during hypoxia the JAK2 /STATs pathway induces XOR activation [54], therefore, it is tempting to speculate that the increase in STAT5 phosphorylation upon allopurinol treatment could be a feedback loop, similar to the one reported for PIM kinases, whose inhibition in combination with TKIs resulted in a synergistic impairment of leukemic cell proliferation [53].

A straightforward consequence of allopurinol treatment is the decrease in intracellular uric acid concentration. Uric acid can react with ROS, NO, and RNS (reactive nitrogen species) triggering oxidation processes that can support cell transformation [55]. Reduction of uric acid level by allopurinol could also contribute to lower intracellular ROS levels, and therefore can be also regarded as an important aspect in the mechanism of action leading to the inhibition of cell proliferation described here. In addition, the existence of ROS independent mechanisms linked to the reduction of cellular uric acid levels cannot be ruled out at this stage, and it will be an interesting aspect to analyze in future studies.

Tumor lysis syndrome (TLS) can be a serious cancer complication derived from excessive cell death, which can eventually lead to organ dysfunction. TLS is characterized by serum hyperuricemia and reducing uric acid levels is regarded as a commonly used prophylactic strategy [56]. Noteworthy, as already mentioned, the use of allopurinol is recommended to minimize TLS associated complications in CML patients [50]. Thus, this strategy could easily be implemented in the clinic and allopurinol could be maintained in the long term, hypothetically increasing the potential achievement of a deep molecular response, one of the main goals in CML treatment.

In the future, it would be interesting to test the combination of XOR inhibitors and TKIs in clinical trials, as well as to test the effect of other recently discovered XOR inhibitors, such as febuxostat, which is more powerful and stable than allopurinol. Its use in the USA has recently been approved by the FDA [57].

## 5. Conclusions

Our results show that XOR inhibition with allopurinol reduces CML cell proliferation and clonogenic capacity. Moreover, the allopurinol and TKIs combinations were significantly more effective than the individual drugs regarding the inhibition of cell proliferation or clonogenic capacity as well as in the induction of cell death. Analysis of drug interaction by the median-effect method as described by Chou-Talalay [24] rendered CIs below 1, supporting the synergistic effect of the TKI plus allopurinol combination on inhibiting cell proliferation. We suggest that these effects are due to the reduction of the intracellular ROS content, which leads to the inhibition of the BCR-ABL signaling cascade. In summary, our results offer a simple, safe, and inexpensive potential therapeutic intervention for CML, which could enhance the effectiveness of TKIs, contribute to the achievement of deep molecular responses, and minimize the possibility of resistance and/or progression.

## Figures and Tables

**Table 1:** Allopurinol and imatinib combination induces cell death in K562 and KCL22 cell lines.

Cell Lines	K562				KCL22			
Content	Control	Allopurinol 0.4 mM	Imatinib 0.2 µM	Allopurinol + Imatinib	Control	Allopurinol 0.6 mM	Imatinib 0.2 µM	Allopurinol + Imatinib
Viable cells	84.6 ± 6.7	78.7 ± 5.6	68.8 ± 5.1 **	49.5 ± 9.3 ***^/+++/###^	91.8 ± 2.0	85.7 ± 2.6 ***	89.7 ± 1.0 *	77.4 ± 2.2 ***^/+++/###^
Early apoptosis	6.3 ± 1.2	12.6 ± 5.0	15.6 ± 4.0 *	27.3 ± 8.1 ***^/++/###^	4.3 ± 0.4	8.8 ± 1.2 ***	6 ± 0.9 **	15.0 ± 2.5 ***^/++/###^
Late apoptosis	2.3 ± 2.6	1.6 ± 0.6	4.8 ± 2.0	5.2 ± 1.5 *^/##^	3.0 ± 1.2	4.5 ± 1.7	3.5 ± 0.6	6.7 ± 1.6 ***^/+/##^
Necrosis	6.8 ± 4.2	7.1 ± 2.5	10.8 ± 2.9	18.1 ± 6.1 ***^/+/###^	1.0 ± 0.8	0.9 ± 0.8	0.9 ± 1.0	0.9 ± 0.8

K562 (*n* = 7) and KCL22 (*n* = 6) cells were treated with imatinib, allopurinol or their combinations. Cell viability was analyzed by flow cytometry staining with Annexin-V/7AAD: viable (Annex^−^/7AAD^−^), early (Annex^+^/7AAD^−^), and late (Annex^+^/7AAD^+^) apoptotic, and necrotic cells (Annex^−^/7AAD^+^). Mean ± standard deviation data are shown. Significant differences: *** *p* < 0.001, ** *p* < 0.01, * *p* < 0.05 with respect to control; ^+++^ *p* < 0.001, ^++^ *p* < 0.01, ^+^ *p* < 0.05 with respect to allopurinol-treated cells; ^###^ *p* < 0.001, ^##^ *p* < 0.01 with respect to imatinib-treated cells.

**Table 2:** Allopurinol and nilotinib combination induces cell death in K562 and KCL22 cell lines.

Cell Lines	K562				KCL22			
Content	Control	Allopurinol 0.6 mM	Nilotinib 50 nM	Allopurinol + Nilotinib	Control	Allopurinol 0.6 mM	Nilotinib 10 nM	Allopurinol + Nilotinib
Viable cells	93.3 ± 1.7	86.1 ± 2.0 ***	65.3 ± 2.4 ***	45.5 ± 3.0 ***^/+++/###^	91.8 ± 2.0	85.7 ± 2.6 ***	82.8 ± 2.2 ***	75.6 ± 2.2 ***^/+++/###^
Early apoptosis	3.7 ± 1.1	8.4 ± 1.5 ***	22.5 ± 1.5 ***	32.1 ± 2.2 **^/++/##^	4.3 ± 0.4	8.8 ± 1.2 ***	10.9 ± 1.9 ***	16.1 ± 2.2 ***^/+++/##^
Late apoptosis	2.2 ± 0.6	4.0 ± 0.7 **	9.9 ± 2.1 ***	19.9 ± 1.9 ***^/+++/###^	3.0 ± 1.2	4.5 ± 1.7	5.6 ± 1.6 **	7.4 ± 2 **
Necrosis	0.7 ± 0.4	1.5 ± 0.3 **	2.3 ± 0.8 **	2.5 ± 0.4 ***^/+^	1.0 ± 0.8	0.9 ± 0.8	0.8 ± 0.7	0.9 ± 0.6

K562 (*n* = 5) and KCL22 (*n* = 6) cells were treated with nilotinib, allopurinol or their combinations. Cell viability was analyzed by flow cytometry staining with Annexin-V/7AAD. Percentage of viable (Annex^−^/7AAD^−^), early (Annex^+^/7AAD^−^), and late (Annex^+^/7AAD^+^) apoptotic, and necrotic cells (Annex^−^/7AAD^+^). Mean ± standard deviation data are shown. Significant differences: *** *p* < 0.001, ** *p* < 0.01 with respect to control; ^+++^ *p* < 0.001, ^++^ *p* < 0.01, ^+^ *p* < 0.05 with respect to allopurinol-treated cells; ^###^ *p* < 0.001, ^##^ *p* < 0.01 with respect to nilotinib-treated cells.
